# The experiences of children with intellectual and developmental disabilities in inclusive schools in Accra, Ghana

**DOI:** 10.4102/ajod.v8i0.542

**Published:** 2019-07-24

**Authors:** Christiana Okyere, Heather M. Aldersey, Rosemary Lysaght

**Affiliations:** 1 School of Rehabilitation Therapy, Queen’s University, Kingston, Canada

**Keywords:** children with intellectual disability, children with developmental disability, children’s experiences, inclusive education, inclusion

## Abstract

**Background:**

Inclusive education is internationally recognised as the best strategy for providing equitable quality education to all children. However, because of the unique challenges they often present, children with intellectual and developmental disabilities (IDDs) are often excluded from inclusive schools. To date, limited research on inclusion has been conducted involving children with IDD as active participants.

**Objectives:**

The study sought to understand the experiences of children with IDDs in learning in inclusive schools in Accra, Ghana.

**Method:**

A qualitative descriptive design was utilised with 16 children with IDDs enrolled in inclusive schools in Accra, Ghana. Participants were recruited through purposive sampling and data were collected using classroom observations, the draw-and-write technique and semi-structured interviews. The data were analysed to identify themes as they emerged.

**Results:**

Children’s experiences in inclusive schools were identified along three major themes: (1) individual characteristics, (2) immediate environments and (3) interactional patterns. Insights from children’s experiences reveal that they faced challenges including corporal punishment for slow performance, victimisation and low family support relating to their learning.

**Conclusion:**

Although children with IDDs receive peer support in inclusion, they experience diverse challenges including peer victimisation, corporal punishment and low family and teacher support in their learning. Improvement in inclusive best practices for children with IDD requires systematic efforts by diverse stakeholders to address identified challenges.

## Introduction

International guiding documents such as the United Nations Convention on the Rights of Persons with Disability and the Sustainable Development Goals emphasise inclusive and equitable quality education for all children (United Nations 2006; United Nations Development Group 2015). Children with disabilities are often denied access to education, particularly in low- and middle-income countries such as Ghana (World Health Organization & World Bank 2011). The situation demands even greater attention for children with intellectual and developmental disabilities (IDDs) who, compared to their peers with other forms of disabilities, record the lowest school enrolment rate (World Health Organization & World Bank 2011).

The Government of Ghana has committed to equal rights to education for children with disabilities enshrined in several of its educational policies. These include article 25 (1) of its 1992 Constitution and Education Strategic Plan of 2003–2015 and 2010–2020 which is committed to include all children with mild to moderate disabilities in mainstream settings (Republic of Ghana 2003, 2012). Despite these provisions, some researchers report that unlike their peers without disabilities, children with disabilities and particularly those with IDDs are discriminated against and institutionalised, with only about 3% of them enrolled in primary education (Ametepee & Anastasiou 2015; Avoke 2002). Children with IDD experience ‘a group of developmental conditions characterized by significant impairment of cognitive functions, which are associated with limitations of learning, adaptive behaviour and skills’ (Salvador-Carulla et al. 2011:177).

The Republic of Ghana’s education system operates on the 6+3+3+4 structure representing 6 years of primary education, 3 years each of junior and senior secondary education and 4 years of undergraduate studies (Education System Ghana 2011; Nketsia 2016). English is the official language of instruction and communication throughout Ghana’s educational system (Education System Ghana 2011). Even though the average recommended class size for primary and secondary schools in the country is 30–35, studies (Alhassan 2014; Kuyini & Desai 2008) report teacher challenges with classroom management because of overcrowded classrooms and lack of resources and services. In particular, children with disabilities often lack the adequate resources and services (i.e. inaccessible curriculum, instructional materials) to succeed in educational systems (Kuyini & Desai 2008).

International human rights documents have recognised the rights of children to express their views and participate in matters that concern them. Similarly, childhood studies theorists argue that children are competent and active social actors ‘with an informed and an informing view of respective social worlds’ (James & James 2004:59), and thus, have the right to be informants in the research that concerns them. Incorporating children as social actors in research requires utilising research techniques such as dialogue and drawings that allow researchers to treat them as equals and understand their experiences and interests (Christensen 2004; James & James 2004).

Although children with disabilities are the biggest stakeholders in inclusion (Bennett, Deluca & Bruns 1997), to date, adult participants without disabilities (i.e. teachers, parents and government officials) have predominated research on inclusion in low- and middle-income countries (i.e. Franck & Joshi 2017; Galovic, Brojcin & Glumbic 2014). Studies in high-income countries that utilised children’s voices in inclusive settings have found that children with disabilities experience challenges including marginalisation (i.e. being shouted at by teachers, peer verbal abuse) and loneliness which negatively impacts their emotions (Adderley et al. 2015; Messiou 2002). Beyond education, studies on children with IDDs have often relied on proxies to collect data, rather than soliciting the views of children with IDD themselves (Majoko 2016; Zachary et al. 2016).

Including children with disabilities as active participants in research on inclusion can provide stakeholders with a unique view of opportunities and challenges and inform targeted practices to support inclusion in the future (Coates & Vickerman 2010). Furthermore, as noted by Rose and Shevlin (2004:160), paying attention to children’s views ‘enable[s] us to reflect upon how future developments may afford greater opportunities to those who have been previously denied’. To that end, we sought to engage children with IDD to learn about their experiences in inclusive schools. Specifically, we collected and analysed the data to answer the following question: what are the experiences of children with IDD in inclusive schools in Accra, Ghana?

## Theoretical framework

The theory that guided our study is the bioecological theory of human development, which was first proposed in the 1970s by Urie Bronfenbrenner (Bronfenbrenner & Morris 1998; Rosa & Tudge 2013). Bronfenbrenner developed this theory to focus research on both the individual and context and understand the complex interrelationship between the individual and environment (Rosa & Tudge 2013). The theory depicts that forces at various levels – biosystem (individual child), microsystem (immediate environment), mesosystem (interactional patterns amongst two microsystems), exosystem (indirect environment), macrosystem (social values) and chronosystem (changes over a period of time) – affect the development of the child (Bronfenbrenner & Morris 1998). Strengths of the theory lie in that it is universally applicable and provides a theoretical and research framework through which both personal characteristics and environmental factors can be factored into the complexities of a child’s development. However, considering the various factors that need to be explored, it is often difficult to achieve hierarchical importance when applying the theory in practice (Rosa & Tudge 2013). However, based on its relevance to an understanding of the personal characteristics and all the contextual factors that influence the inclusion of children with disabilities, we utilised the theory to guide the organisation of the themes that emerged from the data. In this process, we mapped emergent themes onto the various levels of Bronfenbrenner’s bioecological theory of human development.

## Methods

We utilised a qualitative descriptive design as described by Sandelowski (2010), which incorporates overtones or techniques of other qualitative approaches to ensure rigour (Sandelowski 2010). The qualitative descriptive approach stays close to the data and provides a straightforward, rich description and accurate account of the meanings participants ascribe to events (Neergaard et al. 2009). We used this design because it allows flexibility in utilising diverse data collection methods (i.e. observations, drawings and interviews) to derive a detailed account of participants’ experiences about a phenomenon (Sandelowski 2010). We analysed data concurrently with data collection and systematically to identify themes as they emerged. To answer our research question, we used art-based techniques, observations and interviews as the methods of data collection.

### Recruitment

We employed a purposive sampling strategy whereby we approached participants based on specific characteristics such as age, grade, gender and number of years in an inclusive school. We recruited 16 participants from four inclusive schools in Accra, Ghana. Sampled schools were selected through the country’s Special Education Ministry. We identified participants in 14 different classes in the four schools sampled. All participants met the school district’s inclusive education team’s criteria for IDD, which is diagnosed by a screening stage (based on child observations), an achievement test (evidence of academic achievements) and a series of tests conducted in the district assessment centre by a clinical psychologist to confirm the presence of IDD. This classification procedure was facilitated by the district inclusive education team comprising nurses, circuit supervisors and special education coordinators responsible for monitoring and supervising the implementation of school reforms to achieve inclusion in the district (Republic of Ghana 2013). Participants were included in the study if (1) they provided assent and their parents’ consent, (2) they had been in sampled schools for at least a year, (3) they were in the mild to moderate range of IDD and (4) after a review of their available student files including medical reports provided by school heads.

### Data collection

**FIGURE 1:**
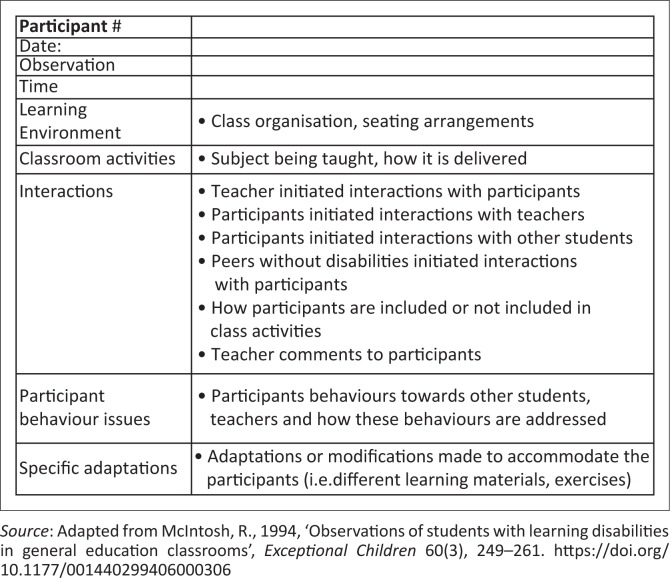
Observation guide.

Data were collected using structured observations, the draw-and-write technique (McWhirter 2014) and interviews. Data collection began with observations in the classroom for an average of 3 hours utilising McIntosh’s (1994) observation categories for students with learning disabilities as a guide (Figure 1 contains the observation guide). Classroom observations and interviews were conducted on the school grounds. Observations included learning environments, teacher and student interactions, teaching adaptations and strategies, and participant behaviours. After each observation, field notes and memos were diarised for later transfer into Microsoft Word files at the end of each day. Writing analytic memos was instrumental in determining concepts requiring further exploration and development (Corbin & Strauss 2008).

To facilitate children’s participation in ways that resonated with them, we utilised the draw-and-write technique described by McWhirter (2014) as an icebreaker to solicit children’s experiences. The technique entails asking children to draw a picture related to a specific topic and write about what is happening in the drawing (McWhirter 2014). Participants were invited to draw images of themselves in school and write about what is happening in the drawing. Participants were encouraged not to be concerned about the outcome of their drawings because their teachers would neither view nor grade them. The results were instrumental in triggering discussions as the task was participatory and participants found it enjoyable and comfortable.

We used semi-structured interviews with participants to build upon emerging themes already identified through observations and drawings and to ask new questions beyond what was observed. (Table 1 details interview questions). Audio-recorded interviews were conducted within 20–25 min of observations, and memos were documented at the end of each day. Writing analytic memos was instrumental to the critical thinking process and in determining concepts requiring further exploration and development (Corbin & Strauss 2008).

**TABLE 1:** Semi-structured interview questions.

Question	Probe or follow-up
Can you tell me about yourself?	What is your name, age, grade, favourites and family?
Can you tell me about your drawing?	Where are you in this picture? What are you doing in your drawing? Who else did you draw? What are these people doing in the drawing? What do you do with the people you drew?
Tell me about school	What is the name of your school? Why do you come to school? What do you do during class period, break or lunch or game time? What do you enjoy most about school? What can be performed to make school more enjoyable for you?
Tell me about your teacher	What is your teacher’s name? What are some of the things teacher can ask you to do in class? Do you get anyone around to help you finish any work teacher gives you? How do you ask your teacher to help? What happens if someone is unkind or hurtful or nasty to you? Do you tell your teacher? What does your teacher do?
How do you find things that will help you do your work?	Do you have textbooks or exercise books for class assignment? Tell me about who helps you to do your work Do you get some help from your friends or teachers? How do they help you? What do they do?
Tell me about your friends	What are the names of your friends? What do you do with your friends in school? Could you ask your friends to help you in class or school? What do your friends do if someone is unkind or hurtful or nasty to you
Do you regard yourself as different from your friends at school?	How do you see yourself similar to or different from your friends?
Do you like this school? Would you want to stay here?	(i.e. segregated special school) If yes, why? If no, why not?
Is there anything else that you would like to tell me?	Anything about teacher, friends, home?

### Ethical considerations

The study was approved by Queen’s University Health Sciences Research Ethics Board in Kingston, Canada (ROMEO/TRAQ: #6019774) and the Ministry of Special Education in Ghana (REF: SE. 183/101). We sought written informed consent from the district and school administrative authorities and parents prior to observations, drawings and interviews and participants provided assent to participate in the interview. We gave participants who could not sign their names properly the opportunity to provide verbal and non-verbal assent. In this process, we asked participants if they were willing to chat about their typical day in school. We included participants who responded yes and in an audible voice or nodded in the affirmative. Pseudonyms were applied to ensure participant confidentiality. We used strategies including reflexivity and member checking to ensure transparency and trustworthiness in the study findings.

## Data analysis

We conducted data analysis alongside observations and interviews (Charmaz 2006). Subsequently, we started preliminary data analysis immediately after the first observation and interview. On a regular basis, the authors shared observations, drawing activities, transcribed interviews and analytic memos with each other during biweekly debriefing sessions for ideas and detailed directions for subsequent interviews. These sessions occurred throughout the data collection period and challenged the authors to think critically about the data while documenting reflections and information relevant to yielding rich data. The debriefing process was also significant to the exposure and critical evaluation of biases and positionality throughout data collection and analyses. Furthermore, the process brought different perspectives to the data and identified emerging themes that formed the basis of additional probes and checks for upcoming observations and interviews (Creswell & Miller 2000).

All observations, interview transcripts, field notes and analytic memos were imported into a computerised qualitative data management software program (Nvivo 2011) to assist in the organisation of data, identification of categories and development of themes. Specifically, we shared the database and analyses of observations and interviews amongst the team. In this process, we became familiar with the data, identified and compared initial codes and grouped similar codes into categories and developed themes central to the purpose of the study.

## Establishing rigour

We employed triangulation, peer debriefing, member checks and reflexivity throughout data collection and analysis to ensure trustworthiness and credibility in the study finding. We triangulated data using several data collection techniques (observations, drawings and interviews) from participants in different school settings. The first two authors engaged in peer-debriefing sessions and explored each other’s views and perspectives (Pandey & Patnaik 2014). We conducted member checks wherein we consistently repeated each participant’s responses during interviews to confirm their agreement prior to asking the next or follow-up questions (Pandey & Patnaik 2014). Additionally, at the end of the data collection process, we organised a closing session where we chatted with participants on emerging themes and key findings. Considering the age of the participants, we used a member-checking approach recommended by Simpson and Quigley (2016) for use with young participants. In this process, we asked participants the same questions asked in previous interviews and compared responses. All of participants’ responses reflected those in previous interviews.

Reflexivity entails researchers consciously examining their biases because of previous experiences, knowledge or connections with the study population (Råheim et al. 2016). Being a Ghanaian, one of the authors approached this study as an insider with the same identity as participants and cultural knowledge of the study context. Further, this author came to this study with an enthusiasm and empathy for children with IDD demonstrated through 5 years’ work for their inclusion in Ghana. Specifically, during this time, the author assisted in enrolling and relocating children with IDD into schools and half-way homes. The author’s insider perspectives, experiences with the study population and the importance attached to their accessing quality education may influence the ability to ask further meaningful and insightful questions and also interpret results from a non-biased culture perspective. Subsequently, the author practised reflexivity wherein there are discussions about the knowledge and experiences of working with children with IDD with the co-authors who recommended strategies (i.e. asking questions in diverse ways, probing for further clarifications) to reduce bias. The authors engaged each other in preliminary data analysis and interpretation of the data, wherein all authors coded these same transcripts independently and came together to compare codes and identify any gaps or divergences in understanding.

## Findings

The primary goal of this study was to understand the experiences of children with IDD in receiving education in inclusive settings in Accra, Ghana. We organised our findings along three main themes: (1) characteristics and struggles at the individual level, highlighting bio- and microsystemic factors, (2) characteristics at the environment level, highlighting macrosystemic factors, and (3) interactional patterns also highlighting microsystemic factors within the Bronfenbrenner’s bioecological systemic framework.

## Participants

A total of eight girls and eight boys participated in the study. Table 2 gives additional information about participant demographics. All students who met the inclusion criteria in a specific class were included in the study. With the exception of one participant who lived with his father and stepmother, all participants lived with both parents. All interviews were conducted in English as participants spoke English in addition to their local dialects.

**TABLE 2:** Participant demographic information.

Name	Gender	Age (years)	Grade	Years in inclusive school
Frank	Male	9	2	1
Lily	Female	10	2	2
Sunshine	Female	10	2	2
Moonlight	Female	10	3	3
Danny	Male	10	3	2
Kofi	Male	11	4	5
Vida	Female	12	4	5
Ivy	Female	13	5	5
Ida	Female	13	4	2
Pearl	Female	13	4	4
Amos	Male	13	4	4
Kwame	Male	14	6	4
Rose	Female	14	4	5
Eric	Male	14	5	5
Evans	Male	15	6	6
Ivan	Male	16	5	5

Note: Pseudonyms were used throughout the study.

### Characteristics and struggles at the individual level – Biosystems

In this section, we present participants’ knowledge and views of themselves in comparison with their peers without disabilities, also covering comments on their personal characteristics (i.e. behavioural challenges) and their effect on their experiences in inclusive schools.

Not all participants explicitly recognised themselves as having an intellectual and/or developmental disability; however, many of them (*n* = 11) stated that they faced academic challenges in school.

‘Madam gives me work but I can’t do it…am never able to finish my work…I like English but when she asks me to read I am not able to read…when she says I should spell, I can’t spell…I don’t understand Math at all…I can understand it but when it gets to exams I do not understand and it worries me.’ (Pearl, 13 years old, School D)

Contrary to most students’ perceptions of similarity to their peers without disabilities, two participants acknowledged individual differences and varying abilities, also highlighting their personal challenges in learning and particularly in examinations. Despite challenges, the participants said that they strived to participate and achieve in the general education classroom. For example, one participant explained:

‘As for the learning it’s different from everyone and what they know…so the little I know I also do it…so we are all different. I am not intelligent but I can write some of the work. I am now learning small things… in exams I don’t do well…I see the thing but don’t know how to write it.’ (Ivan, 16 years old, School D)

Classroom observations indicated that many participants (*n* = 13) exhibited what was considered by the teachers to be ‘behavioural challenges’ (i.e. heads on the table or sleep, slide under desks, engage in fights with peers, look outside classroom window, munch on snacks) during class periods. This was supported by student statements in interviews. For example, one participant reported:

‘When I am tired, I sleep in the class when teacher is teaching…so I don’t do my work or sometimes I will do my work after teacher finish teaching.’ (Lily, 10 years old, School B)

The participants also expressed struggling to maintain attention during class periods, as exemplified in the following quote:

‘I like it when my teacher gives me work in school but when teacher is talking it is difficult to listen and understand so I look in the window and watch the people playing.’ (Danny, 10 years old, School C)

Interestingly, all but one participant blamed the behavioural challenges they experienced on others (i.e. peers and teachers), as noted in the following excerpt:

‘I beat her because she took my pen…she said she will give it to me and she put it there and somebody stole it. I told her to give me my pen but she did not.’ (Kofi, 11 years old, School C)

The participants were often observed to either have their heads on the table or sliding under desks during assignment writing periods and not completing their assignments. Participants who did not complete their assignments sometimes missed out on opportunities to interact with their peers during lunch breaks. For instance, on one occasion, a teacher asked students to visit her desk in turns for an inspection of their assignments and exercise books. She instructed in a loud voice ‘if I do not inspect your exercises you will not step out for break.’ The focus participant in this scenario (Evans, 15 years old, School C) put his head on his table, did not submit his assignment and thus remained in his seat as per the teacher’s order, while his peers vacated the class for their break period.

Despite their learning and behavioural challenges, most participants’ (*n* = 14) responses showed the value they put on school, their interest in learning and expectations for the future. Participants viewed school as necessary for their future successes, achievements and recognition in their respective societies. For instance, one participant noted:

‘I come to school to learn very hard so that when I grow up, I will get work to do and will become somebody in future. When I finish school and they give me certificate I can go to any work and show it to them and they will see that this boy has gone to school… when you stay at home and you don’t go to school, you cannot speak good English and be great in future.’ (Eric, 14 years old, School B)

In as much as participants perceived being educated in their current schools as critical for their future success, they also expressed concern about academics and their need for additional teacher support. In particular, participants expressed worry about not being able to read, write and understand assignments from their teachers and needing assistance from them as highlighted in the following excerpt:

‘I think this school is good for me…no…I don’t want to go to another school. I want to be here…but I want my teacher to help me with spelling and reading and writing. If teacher helps me I can be able to understand…and write. I like this school…I just want someone to always help me to learn.’ (Ivan, 16 years, School D)

### Characteristics at the environment level – Macrosystems

In this section, we present participants’ experiences as they relate to accommodations and modifications in school and home environments:

*School environment*: With the exception of one participant (Vida, 12 years old, School B) whose teacher gave a different assignment (alphabet writing) while other students constructed sentences, there were no explicit modifications and adaptations in instructions, assignments and tests for participants across all observations. For instance, teachers taught for an average of 30 min, followed by a couple of assignments on the board for all students (including participants) to complete within the same duration of time. Further, many participants (*n* = 15) reported using the same materials, textbooks and performing same tasks as other students as exemplified in the quote:

‘Madam gives us the same work to do…sometimes we look on the board and sometimes we look in our class three writing textbook but our work is the same.’ (Eric, 14 years, School B)

Across observations, many participants either submitted assignments upon instruction from the teacher to stop work or continued to write while the teacher moved on to another lesson.

In many of the observed classrooms, teachers disciplined all students (including participants) who turned in assignments after the stipulated time. For instance, for turning in an assignment late, we observed one participant (Kwame, 14 years old, School C) spanked along with other students. In another school and classroom, another participant (Pearl, 13 years old, School D) was not allowed to go for lunch break because of not completing their class assignment.

Additionally, many participants’ desks were found located at the back of the class and out of proximity to their teachers. For instance, on average, a participant’s classroom had five rows of desks with nine desks in each row. Many of the participants in this study were positioned from the fifth to the eighth desk on each row of five. To get to the front of the classroom where their teachers sat or stood to teach, participants walked past five to six desks.

Except for a couple of classrooms that accommodated the exact number of students for its size, we observed that most participants’ classes hosted twice the number of students as there was seating capacity. For instance, one classroom hosted 76 students for a classroom originally designed for 40 students. Subsequently, many participants sat in threes at desks designed for two.

*Home environment*: Some participants talked about receiving support from their families at home relating to completion of homework. In contrast, many other participants (*n* = 10) perceived their families as unsupportive as they did not receive assistance with homework and completed homework either by themselves or returned the work to their teachers uncompleted. Many female participants (*n* = 6) who indicated not receiving assistance with homework also expressed worry about the requirement to do household chores and its impact on their homework and overall learning. For example, one participant reported:

‘When we are learning my mind doesn’t go to the board…I think about the house because when I go home I will fetch water and wash bowls and if I finish I will go and sell so I cannot do my homework.’ (Rose, 14 years, School C)

Some participants (*n* = 9) further discussed not receiving the needed resources from their parents relating to schoolwork and activities. For instance, instead of submitting their assignment for the lesson that was a condition to be permitted to go for lunch break, these participants remained in their seats until their teachers left the class. These participants blamed their lack of participation in class activities and assignments on their parents’ or other relatives’ inability to provide the exercise or textbooks required for participation.

### Interactional patterns – Microsystems

In this section, we present participants’ experiences and perceptions as these relate to their interactions and relations with teachers and peers, also including comments on participation in aspects of school life (i.e. classroom assignments and discussions).

*Teacher interactions*: Students’ perceptions of their teachers were primarily grounded in perceiving the teacher as someone who gives them assignments in the general education classroom. This was exemplified in the following quotes: ‘my madam gives me work in class and I do it’ (Frank, 9 years old, School A), ‘teacher give me plenty work and when I write my hand will be paining me’ (Amos, 13 years old, School A). This basic understanding of a teacher’s role is contrasted by one participant who had a much more multi-faceted and nuanced view of her teachers’ role in her education and overall life:

‘My teacher is a good teacher…my teacher loves me …my teacher likes teaching me every day. My teacher know how to teach …If I do something wrong my teacher will beat me and I will correct myself. My teacher shows me what to do and I will do it. In the classroom, my teacher reads to us and will ask us to say poems….sometimes my teacher can play with us and she laugh with me and when we come to the class she will start teaching us. My teacher gives us homework.’ (Moonlight, 10 years old, School D)

Over half of the participants disclosed the deliberate choices they made to avoid attempts at questions their teachers posed in class because of the fear of being teased by peers for wrong answers.

‘When we are in class and my teacher asks a question, I know the answer, but when I say it some people will laugh because my answer is not correct…so I don’t say anything.’ (Vida, 12 years old, School A)

Across observations, we noticed teachers often engaging with students who volunteered to answer questions. Interestingly, except for a couple of participants who joined in chorus responses and repetition of phrases and sentences, participants were a part of many other students who neither volunteered to answer questions nor were actively engaged during class discussions. After each lesson that lasted, on average, for 30 to 35 min, teachers put assignments on the board and returned to their desks marking registers and exercises.

Participants’ interactions with their teachers revealed their perceptions of them as disciplinarians. For example, participants spoke about their teachers as either spanking or raising their voice at them for not satisfying academic expectations and/or engaging in undisciplined acts as exemplified in the excerpt below:

‘When am not able to answer question madam will cane us or when someone answers madam will say the boy who got the answer correct should take the cane and cane the people who are standing and so when I am standing madam will let the boy cane me.’ (Kwame, 14 years old, School C)

Participants’ perceptions of their teachers matched our findings across observations. For instance, in a couple of instances, teachers were observed spanking all students (including participants) who returned to class from break late.

*Peer interactions*: Many of the participants discussed having cordial and reciprocal relationships with their peers. In particular, participants shared that they loved their peers, playing and working together with them on tasks and extracurricular activities:

‘We love each other…if I am holding something I will give my friends and if they are also holding something they will give me. I do my work with my friends when I come to school, I learn with my friends…we play ball together and at break time… we ran on the park and then we go home together at closing time.’ (Eric, 14 years old, School B)

Many participants (*n* = 13) acknowledged receiving assistance from their peers as it related to classroom assignments as exemplified in the following quote:

‘Sometimes when we go for break and everyone leaves the class, my friend Nat will be with me in the class and I will ask her to teach me. If madam ask me to say something and I don’t know I can tell my friend Andy that…oh Andy this question madam asks I don’t know so please teach me and he will teach me.’ (Kwame, 14 years old, School C)

Much of the time, we observed that participants receive assistance from their peers without disabilities on class assignments, and walk out for break and also travel to their homes with their peers without disabilities. In one instance, a child without disability spent about 25 min helping a participant (Vida, 12 years old, School A) with an assignment.

Across observations, we found many participants sharing desks with their peers without disabilities which allowed for easy and sustained interactions between them. For instance, we identified at least one peer interaction between a participant and their peer without a disability in each of the classrooms in the four sampled schools.

Beyond academics, one participant indicated receiving monetary assistance from her peers:

‘If I don’t have class fees, some of my friends will pay for me, they will pay for me [*class fees*] cost two Ghana cedi’s and sports is one cedi [*equivalent to CAD 1*].’ (Rose, 14 years old, School C)

In spite of positive peer interactions, the issue of bullying was also raised by many participants (*n* = 13) who complained and expressed concern about their peers without disabilities who are either hitting, teasing, insulting or falsely accusing them. For example, one participant reported:

‘They insult me…they are in the class…they beat me…they don’t like me at all…they say that I am dirty and I don’t like dressing…they said my face is like someone who do not speak well. They can insult me that I don’t know anything… they open their mouth to say things that does not make me happy in the class.’ (Ida, 13 years old, School A)

One observation documented that a participant was hit in the back for reasons the culprit was unable to explain to the teacher. Interestingly, one participant reported also being bullied outside school hours:

‘One time one girl started insulting me and she hit me…she said if we close the school she will beat me…so at closing time she hit me and was laughing at me.’ (Ivy, 13 years, School D)

## Discussion

We have presented the main findings of the data from our study that sought to understand the experiences of children with IDD in inclusive schools in Accra, Ghana. We identified three major themes along three levels of Bronfenbrenner’s bioecological theory of human development that shape the experiences of children with IDD in inclusive schools. These are individual characteristics at the biosystemic level, environmental factors at the macrosystemic level and interactional patterns at the microsystemic level. Specifically, we found that personal characteristics (i.e. behavioural challenges), immediate environments (school and home) and occurrences and interactions within these environments influence children’s experiences in inclusive schools.

At the microsystemic level, we found that children with IDD benefitted from peer relations and received support with classroom assignments and travelling to and from school. This finding is consistent with previous studies that have established peer support as beneficial to the academic achievements and social and emotional well-being of children with disabilities (Carter et al. 2005; Franck & Joshi 2017). Specifically, these studies found that educating children with disabilities in inclusive schools provided the opportunity for them to interact and partner with their peers without disabilities on classroom assignments and in travelling to and from school, which also contributed to improving their academic and social interactional skills.

Although participants benefitted from peer support, they were also subjected to verbal and physical abuse by their peers without disabilities during and after school hours. The victimisation of children with disabilities and particularly those with IDD is a persistent social challenge consistent across different countries. For example, in a study in Zimbabwe, Majoko (2016) identified peer bullying and victimisation as a major social barrier to the inclusion of children with IDD. Participants in this study also reported that teachers were not responsive to complaints relating to incidences of bullying and victimisation. This confirms research showing that general education teachers view children with IDD with scepticism and act in an unresponsive manner towards their needs because of the child’s behavioural challenges (Lifshitz, Glaubman & Issawi 2004).

It is noteworthy that children with IDD in general education classrooms are at higher risk for victimisation than their counterparts in segregated special schools. Studies have confirmed that compared to other students without disabilities and their counterparts with other forms of disabilities, children with IDD often experience the highest rates of bullying and victimisation in the general education classroom because of factors including behavioural challenges and lack of social skills (Fisher, Corr & Morin 2016; Sreckovic, Brunsting & Able 2014). Despite peer victimisation, our study suggests an overall positive peer interaction in the general education classroom, indicating that there is no one homogenised narrative of inclusion. Therefore, an in-depth exploration of peer support and victimisation is important for effective inclusive practice. Specifically, future studies should explore potential strategies that can build upon peer support and develop targeted interventions that can be implemented to control the victimisation of children with IDD in general education classrooms. There is a great need for this research, as peer victimisation often results in negative outcomes for students with IDD, including increased behavioural challenges and school dropout (Sreckovic et al. 2014).

Many female participants in this study expressed concern about unsupportive microsystems, and specifically the negative impact of multiple household chores on their school attendance, concentration in class and overall academic competence. Participants discussed feeling tired from tasks such as fetching water, cleaning and selling for their parents resulting in low academic performance. This finding is consistent with research in similar low- and middle-income countries that found girls, without consideration of disability, underperform in school because of multiple chores (Assaad, Levison & Zibani 2010). This study demonstrates that girls with disability are not exempted from the burden of household responsibilities, and indeed, gender seems to be the barrier in this example rather than disability. Girls and women in Ghana are often culturally perceived as primarily responsible for household chores and are often engaged in multiple household chores including cooking (Naami 2015). Further, cultural beliefs and practices within the country privilege boys over girls and thus prioritise their education over their female counterparts (Naami 2015). Compared to their male counterparts, females with disabilities experience multiple forms of discrimination on account of gender and disability (Naami 2015). Subsequently, females with disability are considered a double liability, with often fewer educational opportunities (i.e. low enrolment) and low employment rates (World Health Organization & World Bank 2011). Understanding gender disparities relating to cultural beliefs, attitudes and practices is important for informing best approaches that place priority on females with disabilities as they relate to education support. This is significant to supporting female educational development in countries such as Ghana, where cultural beliefs and practices raise gender issues that impact educational and employment outcomes (Tuwor & Sossou 2017). Future research should explore targeted interventions that can be enacted to protect girls from uneven distribution of household chores and advance their educational development and employment outcomes.

It is widely accepted that modifications in teaching approaches, curriculum and learning environments are critical for achieving the successful inclusion of children with disabilities, including children with IDD (UNESCO 2005). The academic achievements of children with IDD in the general education classroom are dependent on modifications in teaching practices, curriculum provisions and school environments (Chowdhury 2011). Consistent with previous studies in similar low- and middle-income countries (Westbrook & Croft 2015), we found no evidence of differentiated instruction, curriculum accommodations and modifications in learning environments at the macrosystemic level. In all participating schools, children with IDD used the same materials and performed the same tasks as other students in the same time period. Also, the placement of participants’ desks, which put them out of proximity of their teachers, seemed to reduce engagement with them. This is noteworthy as modifications and adaptations in the general education classroom to suit the diverse and unique learning needs and styles of each learner are the underlying principle of inclusive education (Adewumi et al. 2014). Generally, teacher and student interaction in school settings across developing countries is low because of large classroom sizes, low wages and the lack of adequate resources and services (Masino & Niño-Zarazúa 2016). However, compared to their peers without disabilities or with physical and sensory impairments, teachers are sceptical towards children with IDD and interact less with them (Gyimah et al. 2009). This is often attributed to the problem behaviours these children present and the extra instructional skills required to teach them (Gyimah et al. 2009). In a recent study that sought to assess the impact of computer technology on the reading abilities of students with intellectual disabilities in South Africa, Mosito, Warnick and Esambe (2017) found that computer-assisted learning has the potential to advance the academic achievements of students with intellectual disabilities. Our study suggests that inclusive school environments are not accommodating macrosystems for the children with IDD. Given the importance children in this study attached to education as it relates to future aspirations, there is a need for accommodating macrosystems that differentiate teaching and adapt curriculum to satisfy each student’s unique needs. Future studies may seek to understand teacher’s experiences to determine how to improve their capacity to make adaptations to improve inclusive education implementation. Further, we recommend that the Government of Ghana explores the use of computer-assisted learning with children with IDD.

One finding noteworthy of discussion is the use of corporal punishment in inclusive schools. In both observations and interviews, we found that teachers caned participants for not satisfying academic expectations and/or engaging in undisciplined acts. Interestingly, participants revealed that teachers also allowed students who responded correctly to questions to physically punish them for their inability to do likewise. This finding resonates with the study by Agbenyega (2006) which also found teachers using corporal punishment in Ghanaian schools.

The United Nations Committee on the Rights of the Child defines corporal punishment as ‘any punishment in which physical force is used and intended to cause some degree of pain or discomfort, however light’ (United Nations 2007:4). Also, the committee perceives corporal punishment as an act of violence that humiliates and degrades the human dignity of all children. Although international human rights organisations have called for an end to corporal punishment, the practice is still prevalent in many low- and middle-income countries including Ghana (United Nations 2007). In contrast to western societies where the practice has been abolished in school systems (Axelrod 2011), corporal punishment is a culturally acceptable and widely employed method of disciplining children in Ghana. Since its independence, the country has disciplined students using corporal punishment as it is also perceived to motivate learning and train children towards morally upright and responsible adulthood (Agbenyega 2006; Twum-Danso 2013).

The corporal punishment used with children with IDD in this study may, although equitable, be disproportionally targeted. This is because unlike their peers without disabilities, children with IDD are at higher risk of exhibiting problem behaviours (i.e. aggressive and self-injurious behaviours) (Ageranioti-Bélanger et al. 2012) and may be more prone, because of differences in ability, to perform below expected standards. This study shows that although children with IDD have access to inclusive educational opportunities, they are subjected to humiliating school environments or macrosystems, which is a disincentive to their participation and likely to impede their academic and social achievements. Furthermore, as inclusion aims to provide environments supportive of diverse learners (Ainscow & Sandill 2010), inflicting corporal punishment on children with IDD in these settings raises questions concerning stakeholders’ (i.e. teachers, school heads and governments) understanding of disability, inclusion and their willingness to include these children.

Although Ghana has engaged in discussions around prohibiting corporal punishment, the practice persists in many schools across the country as recommendations have not been clearly enacted into laws (Global Initiative to End All Corporate Punishment of Children 2017). As earlier noted, this contrasts with western societies where corporal punishment has been abolished in school systems (Axelrod 2011). Empirical evidence indicates corporal punishment as detrimental to the health, emotional and psychological well-being of all children (Gershoff 2010; Talwar & Carlson 2011). Thus, there is the need to support rather than punish children with IDD for their differences in the inclusive classroom. The Government of Ghana must review policies to eliminate the use of corporal punishment on all children in school settings across the country and integrate monitoring and evaluation systems that reprimand educators for the practice.

Consistent with previous research in similar low- and middle-income countries, some participants discussed their family’s lack of engagement with their education. At the microsystemic level, researchers have attributed family non-involvement and support in the education of their children with disabilities to factors including poverty, lower levels of education and absence of partnerships between key stakeholders such as families and teachers in inclusion (Mapuranga, Dumba & Musodza 2015).

Although this study demonstrates that there is more room for improvement as it relates to teachers implementing inclusion best practices, it is important to note that one key best practice for inclusion is that it requires a systematic effort involving multiple and diverse stakeholders at various levels of Bronfenbrenner’s theory in order to be successful (Majoko 2016). Given that the findings also indicated minimal involvement of families in the education system, one potential opportunity for growth would be to explore family–professional partnerships within the education system. Family–professional partnerships have been recommended as an appropriate strategy to: (1) educate and empower both families and professionals and (2) build substantial and trusting relationships between them as it relates to the inclusion of children with disabilities (Beneke & Cheatham 2016). Thus, it is an important area that needs to be explored in future research in countries such as Ghana where there is minimal family involvement and collaborative partnerships with professionals and, specifically, teachers in inclusion (Kuyini et al. 2016). In addition to families, there is the need for a greater systematic involvement of stakeholders, such as governments, school heads and directors, in the inclusion of children with IDD.

## Limitations

This study was not without limitations. Firstly, because of limited time and resources, children with IDD who participated in this study were selected from four schools in one urban district of the country’s capital that has well-resourced schools compared to rural settings. As inclusive education is practised in other districts, future studies should explore the experiences of children with IDD from other districts in the country where inclusion is practised. Also, selection bias may have been introduced into this study as the district inclusive education team were largely involved in the recruitment of children with IDD. Finally, despite efforts to include children with all levels of IDD in the study, all participants were in the mild to moderate range of IDD. Additional research that explores the experiences of children with severe to profound IDD is needed in order to facilitate a broader view of the issue.

## Conclusion

In this article, we explored the experiences of children with IDD in inclusive schools in Accra, Ghana. We sought to document children’s experiences using classroom observations and interviews. Although participants seemed to benefit from opportunities in inclusive schools, including receiving peer support in learning, they also experience many challenges in learning. The study suggests an overall stressful classroom environment because of large classroom sizes and inadequate supportive measures and resources which makes it challenging for teachers to respond to the additional needs of children with IDD. The study also suggests absence of parental and governmental support in the inclusion of children with IDD. This demonstrates the need for multiple stakeholder action to improve inclusive best practices for children with IDD in Ghana. A commitment of all stakeholders to address the challenges experienced by teachers in inclusive schools would be an important step towards ensuring the full and successful inclusion of all children in inclusive schools in Accra, Ghana.
